# Neutron Reflectivity in Corrosion Research on Metals

**DOI:** 10.1021/acsmaterialsau.4c00011

**Published:** 2024-04-16

**Authors:** Maths Karlsson, Lars-Gunnar Johansson, Laura Mazzei, Jan Froitzheim, Max Wolff

**Affiliations:** † Department of Chemistry and Chemical Engineering, Chalmers University of Technology, 412 96 Gothenburg, Sweden; ‡ Department of Physics and Astronomy, Uppsala University, 75120 Uppsala, Sweden

**Keywords:** neutron reflectivity, surface sensitive, corrosion, oxidation, metals, in situ

## Abstract

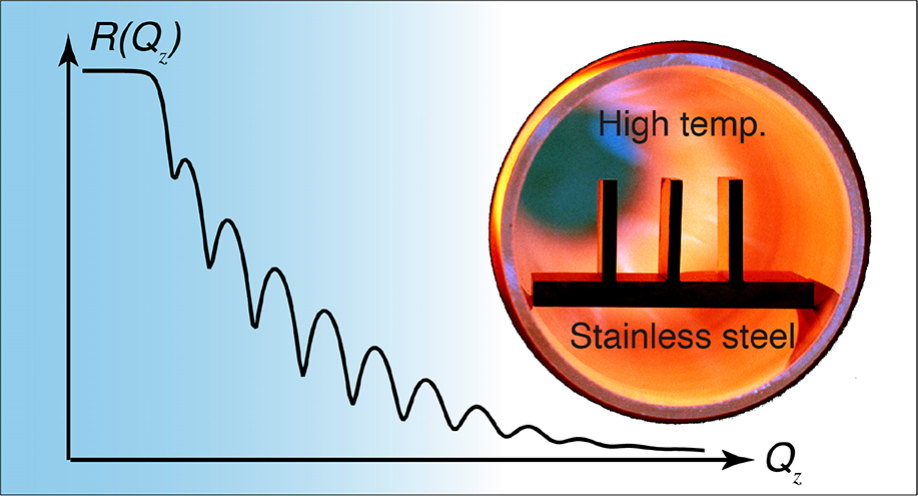

Neutron reflectivity (NR) is potentially a powerful tool for characterizing chemical and morphological changes in thin films and at buried interfaces in corrosion science. While the scope of NR is limited by its inherent demands for low surface roughness and high sample planarity, these drawbacks are compensated for by the unique ability to detect light elements and distinguish between isotopes. Furthermore, the generally weak absorption of neutrons by matter allows the use of bulky sample environments and *in situ* experiments. In particular, the layer thickness range of 3–100 nm accessible by NR is appropriate for studying air-formed films and passive films, which are crucial for the ability of metallic materials to resist corrosion, as well as for investigating the interaction of metal surfaces with hydrogen and its compounds, *e.g.*, water. Also, NR is suitable for studying early stages of oxide growth on metals at high temperature, including the transition from Cabrera–Mott-type films to Wagner-type growth. Here, we outline key characteristics of NR as applied to the study of corrosion of metals, exemplified by earlier work, and discuss perspectives for future work in the field. The aim of our work is to stimulate the application of the unique capabilities of NR to corrosion science.

## 1 Introduction

Corrosion denotes the destructive interaction of a material with its environment by spontaneous chemical reactions.^1^ Metallic materials corrode by reacting with electronegative elements in the environment, forming oxides, hydroxides, chlorides, hydrides, *etc.* The corrosion products often accumulate on the surface but can also form precipitates within the material. Corrosion can cause major damage, *e.g.,* by penetrating vessel walls, destroying the mechanical strength of components, and causing functional materials to fail. It can be life-limiting for components in a jet engine or destroy a precious archeological object in a museum. The cost of corrosion has been estimated to several percent of the gross national product.^1^ Indeed, corrosion challenges the development of new technologies for a sustainable society, *e.g*., power generation with net negative CO_2_ emissions, electrification of industrial processes, and “green” hydrogen production by solid oxide electrolysis. Also, corrosion releases heavy metals into the environment and replacing machinery and other objects destroyed by corrosion increases the demand for scarce metals. The many and diverse corrosion challenges generate a large and growing research in corrosion and corrosion mitigation.

Metallic materials exposed to aqueous media generally suffer *electrochemical corrosion*, whereby the metal is oxidized and dissolved as aqueous ions at anodic sites on the metal surface while the oxidant is reduced at cathodic sites.^1^ The corrosion current is carried by electrons in the solid and ions in the aqueous phase. Many metals tend to form protective, oxide, and hydroxide surface films with a thickness of a few nanometers or less. The presence of such *passive films* drastically reduces the rate of corrosion. In the study of aqueous corrosion, electrochemical methods, such as current/voltage measurements, take center stage by enabling detailed investigation of the corrosion process as it happens, including the anodic and cathodic reactions and passivation and depassivation of the metal. The electrochemical methods are often combined with other *in situ* techniques, e.g., light microscopy or Raman spectroscopy. To investigate the lateral distribution of corrosion and the sensitive relation between corrosion and the microstructure and composition of the metal surface, the samples are subjected to postanalysis by a wide range of analytical techniques.

Corrosion suffered by metals at high temperature (≳300 °C) is another important field. To protect against *high-temperature corrosion*, *e.g.*, rapid carburization, nitridation or excessive oxidation high-temperature alloys are designed to form protective oxide scales, *i.e.*, continuous and slow-growing external oxide layers.^2,3^ Electrochemical techniques are uncommon in high temperature corrosion research due to the lack of a suitable electrolyte. Thermogravimetry is the most important *in situ* method in high-temperature corrosion research. X-ray powder diffraction (XRPD) is valuable in some cases, and the development of *in situ* environmental scanning electron microscopy (ESEM) shows promise. Like aqueous corrosion, high-temperature corrosion is strongly affected by local variations in microstructure and composition at the surface, *e.g.*, grain boundaries, and inclusions. Because of the scarcity of *in situ* techniques, the study of high-temperature corrosion relies heavily on postanalysis methods.

While most investigations of corrosion processes employ gravimetry, optical microscopy, and X-ray diffraction, the number and variety of analytical techniques used for *post analysis* is very large indeed. Electron microscopy (EM) is particularly powerful for imaging and analysis of microstructures and is nowadays applied almost universally in corrosion science. Scanning electron microscopy (SEM), combined with elemental analysis by energy-dispersive X-ray spectroscopy (EDXS), is widely used in both aqueous corrosion and high-temperature corrosion.^4−10^ SEM is used both for imaging the surface and for analyzing cross sections, which are nowadays often prepared by focused ion beam (FIB) milling. The lateral resolution of SEM can reach nanometers. For even higher resolution and for investigating early stages of corrosion, with oxide films in the submicrometer range, transmission electron microscopy (TEM) and scanning transmission electron microscopy (STEM) are applied. Atom probe tomography (APT) can achieve nanometer resolution of the elemental distribution down to parts per million levels in a sample and is becoming increasingly popular in corrosion science.

Early stages of metal corrosion have been studied extensively by, *e.g.*, X-ray photoelectron spectroscopy (XPS)^11^ and Auger electron spectroscopy (AES).^12^ XPS gives information about the elements at the surface and their oxidation states. AES provides elemental composition of the surface with higher lateral resolution than XPS. Infrared (IR) spectroscopy is highly surface sensitive and is used to identify compounds.^13^ Raman spectroscopy provides similar information as IR spectroscopy.^14^ Other post analysis techniques in corrosion science include atomic force microscopy (AFM)^15^ and secondary mass spectroscopy (SIMS).^9,10^ AFM is used for nanometer-resolution imaging and provides topological information. SIMS contributes composition information down to the order of parts per million with high lateral resolution, but matrix effects complicate accurate quantification. It is important to note that many post analysis methods (EM, APT, XPS, AES, SIMS) are vacuum techniques and that artifacts related to sample preparation, radiation *etc*., are common.

In contrast to many of the techniques mentioned above, neutron reflectivity (NR) is sensitive to light elements such as hydrogen. Hydrogen plays a crucial role, especially in aqueous corrosion but also in some cases in high-temperature corrosion. Importantly, NR is potentially very valuable for *in situ* work in a controlled “corrosive” environment. Despite its apparent great potential, NR has been used only very scarcely in corrosion science. Here we outline the key characteristics of NR applied to the study of corrosion of metals, as exemplified by the literature. Rather than providing a comprehensive account of the field, our focus is on the potential for future work. It is hoped that this will stimulate increased use of the NR technique for studies of corrosion of metals.

## 2 Fundamentals of Corrosion of Metals

Exposing a clean metal surface to oxygen gas or dry air results in the dissociative adsorption of O_2_ and charge transfer to generate oxide ions and metal cations, followed by nucleation and lateral growth of an oxide. Once a continuous oxide layer has formed, the oxygen reduction and metal oxidation reactions become separated. At room temperature, thermally activated diffusion of ions in metal oxides is negligible and the ion diffusion necessary for film growth is driven by the large electric field set up across the oxide film by tunneling of electrons to the oxide/gas interface. With increasing film thickness, the tunneling probability rapidly decreases and the electric field is attenuated, explaining why, at room temperature, most metals quickly form an oxide film of 2–5 nm thickness which then ceases to grow.^16,17^ The *native oxide films* formed in ambient air in this manner are decisive for the corrosion behavior of the metals. The *passive films* formed on metals in aqueous solution consist of an inner “barrier” oxide, similar to the air-formed film covered by a layer of hydroxide. Because water can dissolve the passive film, metallic materials tend to suffer *aqueous corrosion*. The solubility of the passive film depends primarily on the aqueous solution (pH, complexing agents) and on the electrochemical potential. The thermodynamics of dissolution and the stability of passive films may be illustrated by potential/pH diagrams.^1^ Because water forms an electrolyte that puts anodic and cathodic regions on the surface in contact, dissolution of the passive film allows electrochemical corrosion, *i.e.*, the anodic dissolution of metal and the cathodic reduction of oxidant occurring simultaneously at different sites. Local breakdown of the passive film triggers many kinds of localized corrosion, *e.g.*, pitting. Many metals are oxidized by water, resulting in the release of hydrogen. In aqueous corrosion, *H*_2_(*g*) tends to form but hydrogen can also dissolve into the metal, giving rise to, *e.g.*, hydrogen embrittlement. Corrosion inhibitors are reactive substances that reduce aqueous corrosion. They are often organic compounds having active groups that form chemical bonds with the surface. The resulting monomolecular film inhibits the metal dissolution reaction (anodic inhibitors) or the cathodic process (cathodic inhibitors).

At high temperatures, thermally activated ion diffusion enables oxide film growth in the absence of a strong electric field. This allows the transport of electrons and ions to continue, driven by the difference in electrochemical potential between the top and bottom of the oxide layer. In a classic paper on “thick” film growth by Wagner,^18^ the case when ionic transport across the oxide layer (with a thickness *X*) is rate-determining was analyzed, showing that the resulting parabolic growth kinetics (d*X*/d*t* = *k*_p_/*X*) are directly related to oxide properties, *i.e.*, ion diffusivities.^2,3^ The resulting *oxide scales* may reach >100 μm in thickness. The slow-growing *protective oxide scale* is ideally single phase and must adhere to the metal, be unreactive, and have low vapor pressure. Also, to be selectively oxidized, the element forming the protective scale must have greater affinity for oxygen than the other main elements in the alloy. Only a handful of oxides are used to form protective oxide scales on technologically relevant alloys, the most important being Cr_2_O_3_ and α-Al_2_O_3_.^2,3^ The formation of a protective scale on an alloy requires that transport of the oxide-forming element to the alloy surface is fast enough to keep up with the growth rate of the protective oxide. Otherwise, the desired oxide precipitates within the alloy = *internal oxidation*, rather than forming a protective surface layer, triggering rapid corrosion.^2,3^

## 3 Neutron Reflectivity

Neutrons interact with the nuclei, and the strength of the interaction is quantified by the coherent neutron scattering length, *b*_coh_, for the different isotopes. In contrast, absorption and incoherent scattering are often of minor importance for NR. The attenuation of the beam is low and not considered here. *b*_coh_ varies irregularly across isotopes as well as in the periodic table. This is very different from X-rays, which scatter from electrons, and hence, the scattering increases systematically with atomic number; Table 1 compiles *b*_coh_ and *f*, the equivalent interaction for X-rays, for a selection of elements and isotopes. Importantly, neutrons can be very sensitive to light elements, which may be difficult or even impossible to detect with X-rays. Further, isotopes of the same element or elements with similar atomic numbers can feature very different *b*_coh_ values and hence may show great contrast in neutron scattering data. Crucially, neutrons are only very weakly absorbed by most elements, *e.g.*, aluminum, enabling the use of complex sample environments.

**Table 1:** Coherent Neutron Scattering Length *b*_coh_ and Electronic Scattering Factor *f*, for the Hydrogen Isotopes ^1^H and ^2^H (D), O, Cr, and the Two Fe Isotopes ^56^Fe and ^54^Fe

	^1^H	^2^H	^16^O	^52^Cr	^56^Fe	^54^Fe
*b*_coh_ (fm)	–3.7406	6.671	5.803	4.920	9.94	4.2
*f*	0.99	0.99	7.99	23.84	24.86	24.86

**Figure 1:**
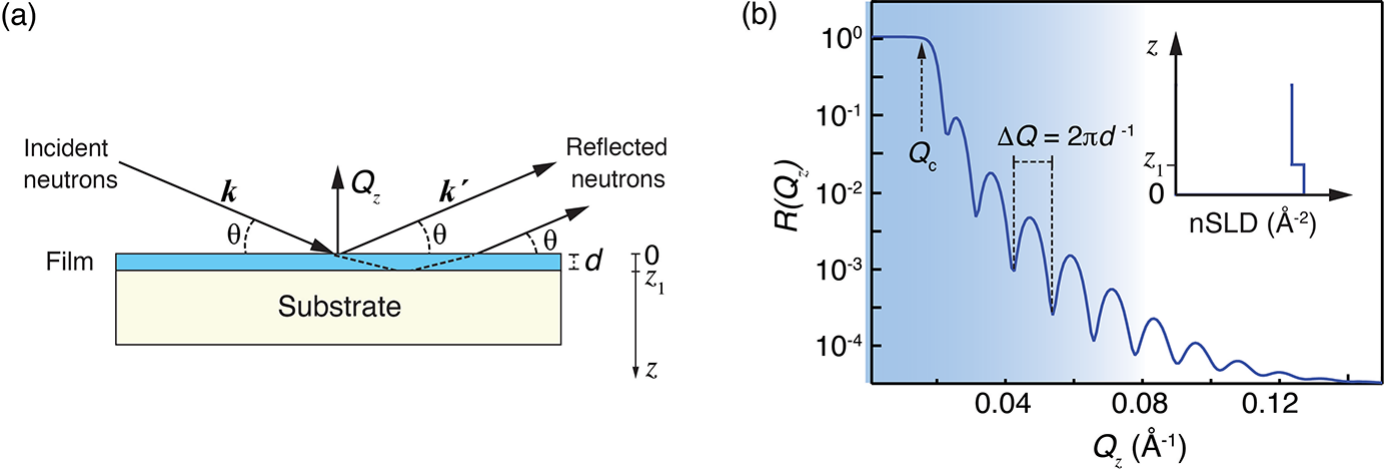
(a) Geometry for specular NR from a sample consisting of a thin film (of thickness *d*) on top of a substrate. (b) Example reflectivity profil and corresponding *nSLD* (inset) for the sample in (a). The critical edge (*Q*_*c*_) and the period of the Kiessig fringes, which relates to the film thickness as Δ*Q* = 2π*d*^*–1*^, are indicated.

The details of the NR technique, which is designed to study the structure of thin films and other layered systems, can be found elsewhere^19^ and recently a review on the study of surface coatings with NR was published.^20^ Shortly, the technique is based on the measurement of the reflected intensity *R* of a neutron beam sent under grazing incidence on the surface to be studied. A sketch of the scattering geometry is shown in Figure 1a. The neutrons impinging on the sample are characterized by their wavelength λ = 2π/***k****,* where ***k*** is the wavevector of the incident neutrons, and the (incident) angle θ between the beam and the sample surface. Here we only consider specular reflection, as illustrated in the panel, meaning that the angle of reflection is equal to the incident angle. Moreover, only elastic scattering is considered, |***k’***| = |***k***|, with ***k’*** being the wavevector of the reflected neutrons. The transferred momentum ***Q*** is oriented in the direction *z* along the sample normal, with **Q***_z_* = ***k’*** – ***k*** = 4π sin θ/λ. The quantity obtained in an NR experiment is the reflected intensity *R* as a function of ***Q***_*z*_.

*R*(*Q*_*z*_) can be calculated from the neutron scattering length density (*nSLD*) profile along the sample surface normal. *nSLD* is defined as
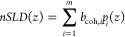
where *p*_*i*_(z) is the number density of each isotope (*i*) present at a certain depth in the material, weighted by its coherent neutron scattering length *b*_coh,*i*_. In practice, a layered model system (*nSLD* profile, as shown in the upper right insert of Figure 1b) is constructed and *R* is calculated. This model is then refined by fitting. By applying constraints from complementary experimental methods, we then chose the best *nSLD* model to describe the investigated material. In this context, X-ray reflectivity (XRR) measurements are particularly useful as they probe the electron density profile on the same length scale but are readily available in the lab or of much higher brilliance, if synchrotron radiation is applied. Note, specular NR is only sensitive to the *nSLD* profile along the surface normal but only indirectly to its lateral fluctuations. If lateral (in-plane) correlations need to be probed the method must be extended to off-specular and grazing incidence small angle scattering (GISANS). For more details on these techniques, we refer to ref. (21).

Figure 1b shows simulated NR for a homogeneous, flat, film of thickness *d* on a substrate. The interference fringes (known as Kiessig fringes) resulting from the interference of neutrons being reflected at the top and bottom of the layer are well visible. Their amplitude and width are defined by the difference in *nSLD* of the film with respect to the vacuum and the substrate and the thickness of the film, respectively. At larger ***Q*_*z*_** the film thickness can be directly calculated from the frequency of the Kiessig fringes by *d* = 2π/Δ*Q*. At low ***Q*_*z*_** values, all intensity is totally externally reflected. At a critical value, ***Q*_c_**, which is defined by the *nSLD* of the sample, according to , the reflectivity decays steeply as ***Q*_*z*_** increases. For relatively large ***Q*_*z*_** values, this decay converges toward ***Q*_*z*_**^–4^. Roughness will result in a steeper decay of the reflectivity with ***Q*_*z*_** and the rougher the interface, the steeper the decrease will be. This allows measurement of the roughness of the interfaces.

As the signal-to-noise ratio of measurements is limited, typically 10^–4^–10^–7^, only relatively flat interfaces, with a roughness typically less than some nm, can be studied. For more than one layer, the reflections of neutrons at all the interfaces have to be considered and the interference needs to be calculated but the basic principle remains as described above.

## 4 Case Studies

NR has been applied to study oxidation, corrosion and adsorption of corrosion inhibitors on a range of metal surfaces, including Fe,^22−27^ FeCr alloys and stainless steel,^28,29^ Ni and Ni alloys,^30−33^ Co,^25^ Cu,^33−35^ Ti,^25,36−40^ Zr,^41−43^ and Al.^44−46^ For a more comprehensive coverage of the literature, see reviews in refs (47) and (48).

### 4.1 NR Studies of Passive Film Growth on Metals in Aqueous Solution

Several NR corrosion studies deal with aqueous systems, investigating passive films on metals.^28,31−33,35,37,38,40−44,46^ Much of this work focuses on changes in the morphology and composition of the surface layers induced *in situ* by electrochemical polarization and solution pH. The experiments generally involve thin metallic films deposited on highly flat, neutron-transparent substrates, typically single crystal silicon. To investigate the solid–liquid interface while avoiding incoherent scattering from the electrolyte, the neutron beam, which is reflected from the electrode-liquid interface, enters from the substrate-side. For more details on this scattering geometry, we refer to a recent review.^49^ On certain metals, *e.g.*, Ti, Zr, and Al, the thickness of the passive film tends to be proportional to the degree of anodic polarization. The relatively thick, so-called *anodized films* that can be generated in this manner are commonly used for corrosion protection.

Wiesler et al.^39^ used *in situ* NR to study passive film formation on Ti thin films on Si. The films were immersed in H_2_SO_4_(aq) and subjected to anodic polarization. Hydrogen was reported to be incorporated into the Ti metal during the resulting TiO_2_ film growth. Tun et al.^37,38^ report that the film formed during anodic polarization consisted of a bottom, TiO_2_, part and an outer, hydrated oxide. Cathodic polarization did not change the film thickness but resulted in hydrogen incorporation into the film and, eventually, hydrogen uptake by Ti. Vezvaie et al.^40^ studied the uptake of deuterium by Ti during cathodic polarization, by simultaneous NR and electrochemical impedance spectroscopy (EIS). They report that the passive film failed beneath a threshold cathodic potential as TiO_2_ was reduced to TiOOD (TiOOH), causing hydrogen to penetrate the metal. Film failure was preceded by the onset of electronic conductivity.

A similar approach was used to study passive films on Zr.^41−43^ While the thickness of anodic ZrO_2_ films on Zr was proportional to the anodic polarization, similar to Ti, increasing the anodic voltage above a threshold value caused cracking of the (12 nm) ZrO_2_ film, resulting in the loss of passivity. Water in the cracks decreased the film *nSLD* and reacted with Zr, causing hydrogen to dissolve in the metal substrate. In contrast to Ti, cathodic polarization of Zr did not result in hydrogen uptake by the oxide film or by the metal substrate. Hu et.al^44^ investigated anodized films on aluminum by NR. Such films consist of a dense Al_2_O_3_ inner region and a porous outer region and have to be treated in order to seal the pores. NR was used to help elucidate the morphological and chemical changes induced by such treatments. Ha et al.^28,31^ used PNR to investigate the effect of applied potential on passive film growth in alkaline solution on a Fe(80%)-Cr(20%) alloy and on nickel, respectively. Both metals were deposited on Si substrates. The film thickness was proportional to the applied anodic potential in both cases. For the FeCr alloy, they concluded that the passive film consisted of two layers, an inner region consisting of Cr_2_O_3_ or FeCr_2_O_4_, and an outer Cr(OH)_3_ region. In the case of nickel, the film consisted of NiO.

Cwalina et al.^32^ and C. Lutton et al.^33^ used NR for *in situ* studies of passive film formation and dissolution on NiCr and NiCrMo alloys in chloride- and sulfate-containing solutions, respectively. In both investigations, NR was complemented by electrochemical techniques and by several other analytical methods to enable a more comprehensive understanding. NR showed that the passive films consisted of a bottom oxide part covered by a layer of hydrated oxide. Ni-rich films formed early during the passivation process, while Cr(III) enrichment was observed at longer times. The concentration of Cr^3+^ in the film increased at low pH and in chloride solution. Alloying with Mo increased the amount of chromium in the passive film. Situm et al.^35^ used NR to investigate the hydrogen uptake by a two-layered film consisting of 50 nm Cu on 4 nm of Ti on single crystal Si. Cathodic polarization of the film in NaCl(aq) solution at pH 9 resulted both in hydrogen evolution and in hydrogen pick up by the duplex metal film. Thus, hydrogen accumulated in the Ti sublayer while the amount of hydrogen in copper was below the detection limit.

### 4.2 NR Investigations of the Interaction of Molecules with Metal Surfaces and the Formation of Anticorrosive Films

NR has been used in several studies to investigate the interaction of molecules with metal surfaces and the formation of anticorrosive films.^22−24,26,29,30,34,45,50^ In most cases, the NR measurements are not complemented by electrochemical methods. Wood et al.^22−24^ and Poon et al.,^26^ used PNR, in combination with XRR and other techniques, to study the interaction of candidate corrosion-inhibiting surfactants with oxide-covered Fe films on Si. While it was suggested in^24^ that bis(2-ethylhexyl)phosphate was chemisorbed on the surface, it was later concluded that the surfactant reacted with the surface to form a layer of iron(II) phosphate.^26^ Wood et al.^30^ used PNR to study the interaction of surfactants with Ni films on silicon. It was found that the anionic sodium dodecyl sulfate was chemisorbed on the Ni surface and that it afforded considerable corrosion protection in acidic solution, even at submonolayer coverage. Using PNR to investigate the interaction of a CuO-covered Cu film on Si with surface active compounds, Welbourn et al.^34^ reported that hexadecylamine formed a chemisorbed surface layer which protected against sulfidation to some extent. Investigating the interplay between stainless steel implants and body fluids, Wood et al.^29^ used PNR to study the interaction of fibrinogen with a stainless-steel film deposited on Si. The successful deposition of an FCC-structured FeCrNi film is noteworthy. However, the binding of the protein molecule to the passive-film-covered surface could not be determined. Investigating spin-coated vanadate-based corrosion inhibitor films on an aluminum alloy by NR and XRR, Wang et al.^45^ report that the bottom part of the resulting film was dense and hydrophobic while the outer part was porous and absorbed water. Investigating bioinspired organic polymer coatings intended for corrosion protection of various metals, Payra et al.^50^ used NR to study film formation on bulk silicon. The measurements showed the formation of dense, 5 nm thick layers on the surface.

### 4.3 NR Studies of Oxide Film Formation on Metals in Air and in Other Gases

Relatively few NR investigations focus on surface films formed in air under ambient conditions.^25,27,36,51,52^ The air-formed film on iron was recently investigated by NR and PNR supported by XRR.^27^ Iron was deposited on quartz, providing a smooth surface. The reflectivity data showed that the air-formed iron oxide film was 3 nm thick, uniform, and fully dense and it was concluded that it consisted of magnetite (Fe_3_O_4_) rather than maghemite (γ-Fe_2_O_3_) or hematite(α-Fe_2_O_3_). However, the oxide film did not exhibit the (ferri)magnetic properties characteristic of magnetite. This was ascribed to the thinness of the air-formed film. Watkins et al.^51^ followed the formation of a passivating oxide layer on U(92%)-Nb(6%) alloy films, by NR and XRR measurements, both at early oxidation and after one year. Combined analyses by NR, XRR, SEM/EDX, and X-ray diffraction revealed that the film consisted of a 30–35 nm UO_2_ top-layer and an inner Nb_2_O_5_ layer. The overall concentrations of U and Nb in the oxide were roughly equivalent to those in the alloy, indicating that preferential oxidation did not take place. Mongstad et al.^52^ used NR to investigate metallic and semiconducting YH_*x*_ films deposited on a Si substrate and observed the formation of a 5–10 nm surface oxide layer in ambient air.

The virtual absence of NR studies on the formation of oxide films at high temperatures in the literature is attributed to experimental difficulties discussed below.

## 5 Critical Remarks

As shown by the examples, NR is a nondestructive tool to characterize the thickness, roughness, and composition of thin layers on metal surfaces which can give important information about corrosion processes. A particular advantage associated with NR is the significant scattering from light elements, including H and Li, as well as its isotope dependence on the same elements, which makes studies of very thin adsorbed layers at buried interfaces possible. Specifically, the accumulation of H at a relatively flat scale/alloy interface is detectable in NR because of the associated change in the *nSLD*. Exchanging H for D provides additional data, strengthening the interpretation of results. These unique features of NR allow to perform *in situ* measurements of (H/D rich) layers that form beneath an existing oxide scale. Indeed, few if any other techniques can achieve *in situ* detection of such sublayers. Also, because of the labile nature of H, post analysis by itself can hardly provide data to test hypotheses relating to the role of H in oxidation and corrosion processes.

A major limitation of NR in corrosion science is that the samples under investigation need to be very flat. While atomically smooth samples may be prepared using thin film deposition techniques, subsequent oxidation/corrosion likely leads to increased surface roughness, affecting the quality of the reflectivity signal. Additionally, NR requires a relatively large surface area (typically >100 mm^2^). In general, the large surface area required limits the use of NR in this field because of the localized nature of many critical corrosion processes. Many important types of aqueous corrosion, *i.e.*, involving the breakdown of passive films, are localized on length-scales down to 100 nm. The lack of lateral resolution also restricts the use of NR in high-temperature corrosion, especially for alloys and in complex environments. Off-specular and GISANS may be good techniques to overcome some of these challenges,^21^ but they may be hard to observe. The measuring time for one data set is typically on the order of minutes to several hours. This means that relatively fast processes, such as rapid oxide growth on a metal, are challenging to observe *in situ* with NR. Quenching the samples at different oxidation states can help overcome these challenges.

Passive films play a major role in the corrosion chemistry of stainless steels and for, *e.g.,* Zr, Ti, Al and their alloys, *cf.*Section 1. Importantly, the thickness and properties of passive films can be controlled *in situ*, during the NR experiment, by anodic and cathodic polarization and, *e.g.*, by solution pH. Moreover, electrochemical characterization, providing essential information on the electric properties of the films, can also be applied *in situ*, *cf.*Section 4.1. For similar reasons, NR is well adapted for investigating inhibitor films, *e.g.*, on steel surfaces in aqueous solution, *cf.*Section 4.2. It is noted that these NR studies relate to the situation before the onset of substantial corrosion. In contrast, NR is not easily applicable to “regular corrosion”, both because surfaces become rough and since corrosion attack typically varies laterally over the surface, *cf.*Section 1.

This review shows that much of the corrosion-related NR work uses thin metal films deposited on, *e.g*., silicon. While such metal films are well-suited for reflectometry by providing highly flat interfaces, the microstructure, composition and properties of the films are substantially different from bulk metals, especially in the case of alloys. Therefore, it is desirable to carry out NR experiments also on oxide layers and other films on the surface of bulk metals and alloys. NR corrosion work in the absence of an aqueous electrolyte is scarce and mainly concerns oxide films formed in ambient air.

**Figure 2:**
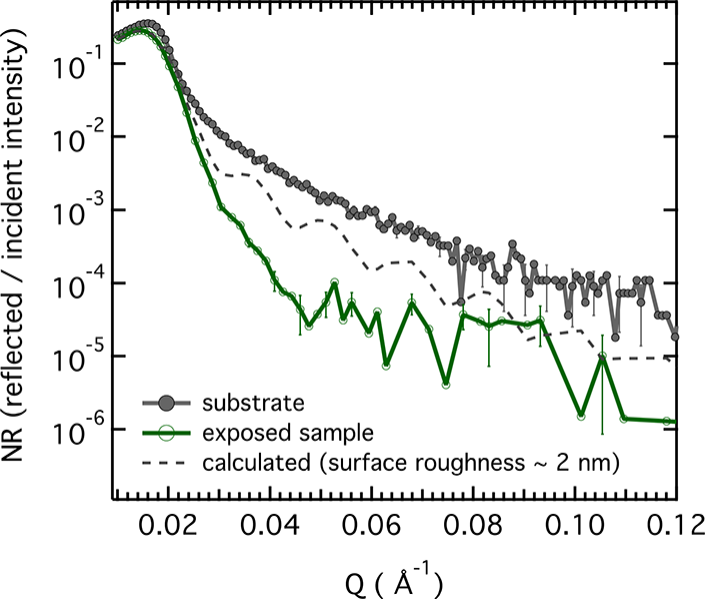
NR curves as measured on polished Fe-20%Cr alloy. The as-polished substrate (black line) and sample that was exposed to 20 min at 800 °C in air (green line). A calculated curve for the case of a sample with low roughness (gray, dashed line) is also shown. The oxidation treatment was carried out at Chalmers University of Technology. The NR measurements were done at the Swedish Super ADAM beamline at the Institut Laue-Langevin in Grenoble, France. (Unpublished.)

Figure 2 shows NR data measured on an FeCr alloy in sheet metal form. The alloy is intended for use as a bipolar plate in solid oxide fuel cells and solid oxide electrolysis cells. During operation at 600–800 °C, the bipolar steel plate is exposed to H_2_ on the fuel side and air on the other side. The presence of H_2_ is known to damage the spontaneously formed protective oxide layer on the air side, which can lead to failure of the device.^6,9^ While the “dual atmosphere effect” is apparently coupled to hydrogen dissolved in the alloy, which compromises the alloy′s ability to form a protective chromia layer, little is known about the underlying mechanism. To elucidate this long-standing problem, the viability of NR was studied. The data in Figure 2 compare the as-polished steel with a sample that was polished and subsequently oxidized at 800 °C in air. Analysis of the NR data shows that the exposure leads to an increase in surface roughness from ≈1 nm (on the surface of the substrate) to ≈5 nm. Furthermore, it indicates that the ≈5 nm roughness is mainly at the oxide/air interface, meaning that the low roughness at the substrate/oxide interface is virtually unaltered under exposure. The increase in surface roughness causes a damping of the oscillations of the NR pattern; see, *e.g.*, the calculated NR curve in Figure 2. This effect makes the extraction of the chemical composition and quantification of H in the film very difficult. These challenges may be overcome to some extent by improving sample preparation, *i.e*., by minimizing sample deformation during exposure.

## 6 Outlook

There is clearly a wide scope for NR studies to explore many aspects of the corrosion mechanisms of metals. In order to be studied by a NR experiment, surface films must be macroscopically homogeneous (cm^2^ scale), flat (<3 nm roughness), and thin (3–100 nm). If these requirements are fulfilled, NR can provide valuable information which complements other, more conventional analysis techniques.

The “dual atmosphere effect” mentioned above shows that hydrogen can play a major role in high temperature corrosion and there are many other such examples, often in cases where H_2_O(g) is the oxidant.^10,53^ However, the difficulty of detecting hydrogen by postanalysis has frustrated research in this field. While the method is restricted to rather thin oxide layers, the ability of NR to perform *in situ* analysis and detect hydrogen in a surface oxide or beneath it can provide a valuable new experimental approach in this field. The problems encountered concerning sample planarity imply that work with bulk metals may have to be complemented by measurements on thin metal films. In that case, the thermal expansion mismatch between substrate and the film must be considered.

In high-temperature oxidation research there is a scarcity of information on protective oxide layers in the 5–100 nm range. While most protective oxide scales encountered in practice are >500 nm thick, thinner oxide layers represent early stages of growth which may be decisive for the morphology and adhesion of the layers. Moreover, the film thickness range accessible by NR corresponds to the little studied transition from (low temperature) field-driven oxide film growth to Wagner-type growth of thick films.^2,17^ It is suggested that NR could make important contributions to understanding early oxide film growth on pure metals, including the roles of hydrogen and water.

In most cases, the formation of a protective oxide scale at high temperature involves an initial stage, where oxygen reacts indiscriminately with the alloy components, forming a mixture of oxides on the surface. The “mixed oxide” layer grows rapidly in thickness, resulting in a drop of oxygen activity at the metal-oxide interface, which causes the oxidation of the more noble alloy components to cease. Simultaneously, the more oxygen-active element in the alloy tends to react with the less stable oxides in the initial oxide layer, *e.g.*, 2Cr(alloy) + 3NiO(s) → Cr_2_O_3_(*s*) + 3Ni. The metal produced in this reaction may re-enter the alloy or form particles on the oxide scale. This process is termed *transient oxidation* and ends when a continuous protective oxide layer has formed at the oxide/metal interface.^2^ While transient oxidation is decisive for the ability of an alloy to resist high-temperature corrosion, it is poorly understood, partly because of limitations in the available techniques. It is suggested that NR experiments may help understand the development of protective oxide layers on single-phase alloys, *e.g.*, investigating the porosity which tends to appear in the metal, immediately beneath the oxide layer, because of the rapid consumption of an alloy component (*i.e.*, Cr or Al).

*Atmospheric corrosion* is a special case of aqueous corrosion where the use of electrochemical methods is limited because of the small amount of electrolyte present.^54^ The capacity of NR to differentiate between H and D allows *in situ* atmospheric corrosion experiments. One suggestion is to study the pick-up of H by metals, *e.g.*, in the case of magnesium where the role of hydrogen is much debated.^55^ The experiment would involve comparing NR measurements carried out in H_2_O(g) and D_2_O(g) environments, respectively. The experiment requires the use of a sample compartment, which allows control of the gas composition, *i.e.*, relative humidity, H_2_O/D_2_O, etc. Also, NR may be used in a similar way to investigate other aspects of atmospheric corrosion, including the buildup of surface electrolyte and the early growth of protective surface films such as layered double hydroxides.

## 7 Conclusions

Fundamental and applied research on the oxidation and corrosion of metals and alloys has a key role in the development of new functional materials for many applications. This Perspective demonstrates that NR is a powerful *in situ* technique which can contribute toward understanding several aspects of corrosion and corrosion protection which are difficult to study by other techniques. Even more importantly, it points toward new opportunities for research in this field. Further research in this field is likely to include studies of the effect of hydrogen/water on corrosion of metal and metal alloys, and the exploitation of advanced sample environments for *in situ* studies, such as under controlled environments and as a function of temperature. It is noted that, as new neutron sources with higher beam intensities, such as the European Spallation Source, become available, the limitations of NR will become mitigated to some extent. It is concluded that NR is far from being used to its full potential in this area of research.

## References

[ref1] McCafferty E. Introduction to Corrosion Science, 1st ed.; Springer-Verlag: New York., 2010.

[ref2] Kofstad P. High temperature Corrosion; Elsevier Applied Science: London, 1988.

[ref3] Young D. J. High Temperature Oxidation and Corrosion of Metals, 2nd ed.; Elsevier, 2016.

[ref4] Dillmann Ph.; Mazaudier F.; Hœrlé S. Advances in understanding atmospheric corrosion of iron. I. Rust characterisation of ancient ferrous artefacts exposed to indoor atmospheric corrosion. Corros. Sci. 2004, 46, 1401–1429. 10.1016/j.corsci.2003.09.027.

[ref5] Esmaily E.; Zeng Z.; Mortzavi A. N.; Gullino A.; Choudhary S.; Derra T.; Benn F.; Délia F.; Müther M.; Thomas S.; Huang A.; Allanore A.; Kopp A.; Birbilis N. A detailed microstructural and corrosion analysis of magnesium alloy WE43 manufactured by selective laser melting. Additive Manufacturing 2020, 35, 101321 10.1016/j.addma.2020.101321.

[ref6] Gunduz K. O.; Chyrkin A.; Goebel C.; Hansen L.; Hjorth O.; Svensson J. E.; Froitzheim J. The effect of hydrogen on the breakdown of the protective oxide scale in solid oxide fuel cell interconnects. Corros. Sci. 2021, 179, 109112 10.1016/j.corsci.2020.109112.

[ref7] Eklund J.; Persdotter A.; Hanif I.; Bigdeli S.; Jonsson T. Secondary corrosion protection of FeCr(Al) model alloys at 600 °C – The influence of Cr and Al after breakaway corrosion. Corros. Sci. 2021, 189, 109584 10.1016/j.corsci.2021.109584.

[ref8] Mortazavi A. N.; Esmaily M.; Geers C.; Birbilis N.; Svensson J. E.; Halvarsson M.; Chandrasekaran D.; Johansson L. G. Exploring failure modes of alumina scales on FeCrAl and FeNiCrAl alloys in a nitriding environment. Acta Mater. 2020, 201, 131–146. 10.1016/j.actamat.2020.09.058.

[ref9] Falk-Windisch H.; Malmberg P.; Sattari M.; Svensson J. E.; Froitzheim J. Determination of the oxide scale growth mechanism using 180-tracer experiments in combination with Transmission Electron Microscopy and nanoscale Secondary Ion Mass Spectrometry. Mater. Charact. 2018, 136, 128–133. 10.1016/j.matchar.2017.12.001.

[ref10] Mortazavi N.; Geers C.; Esmaily M.; Babic V.; Sattari M.; Lindgren K.; Malmberg P.; Jönsson B.; Halvarsson M.; Svensson J. E.; Panas I.; Johansson L. G. Interplay of water and reactive elements in oxidation of alumina-forming alloys. Nat. Mater. 2018, 17, 610–617. 10.1038/s41563-018-0105-6. 29891892

[ref11] Strehblow H. H.; Marcus P. In Analytical Methods in Corrosion Science and Engineering; Marcus P., Mansfeld F.; Eds.; CRC Press, 2006.

[ref12] Castle J. E. In Analytical Methods in Corrosion Science and Engineering; Marcus P., Mansfeld F., Eds.; CRC Press, 2006.

[ref13] Leygraf C.; Johnson M.. In Analytical Methods in Corrosion Science and Engineering; Marcus P., Mansfeld F., Eds.; CRC Press, 2006.

[ref14] Rostron P.; Gaber S.; Gaber D. Raman Spectroscopy. Review. Int. J. Eng. Res. Technol. 2016, 6, 50–64.

[ref15] Maurice V.; Marcus P. Progress in corrosion science at atomic and nanometric scales. Prog. Mater. Sci. 2018, 95, 132–171. 10.1016/j.pmatsci.2018.03.001.

[ref16] Cabrera N.; Mott N. F. Theory of the oxidation of metals. Rep. Prog. Phys. 1949, 12, 163 10.1088/0034-4885/12/1/308.

[ref17] Atkinson A. Transport processes during the growth of oxide films at elevated temperature. Rev. Mod. Phys. 1985, 57, 437–470. 10.1103/RevModPhys.57.437.

[ref18] Wagner C. Beitrag zur theorie des anlaufvorgangs. Z. phys. Chem. 1933, 21B, 25–41. 10.1515/zpch-1933-2105.

[ref19] Penfold J. Principles of reflectometry with reactor and pulsed sources. In Neutron Reflectometry - A Probe for Materials Surfaces; International Atomic Energy Agency: Vienna, Austria, 2006.

[ref20] Wolff M.; Gutfreund P. Neutron reflectivity for the investigation of coatings and functional layers. In Handbook of Modern coating technologies, Advanced characterisation methods; Aliofkhazraei M., Nasar A., Chipara M., Laidani N., De Hosson J. Th. M., Eds.; Elsevier, Amsterdam, Oxford, Cambridge, 2021; Vol. 2, pp 143–175.

[ref21] Wolff M. Grazing indicent scattering. EPJ. Web of Conferences 2018, 188, 04002 10.1051/epjconf/201818804002.

[ref22] Wood M. H.; Casford M. T.; Steitz R.; Zarhakhsh A.; Welbourn R.; Clark S. M. Comparative Adsorption of Saturated and Unsaturated Fatty Acids at the Iron Oxide/Oil Interface. Langmuir 2016, 32, 534–540. 10.1021/acs.langmuir.5b04435. 26707597

[ref23] Wood M. H.; Welbourn R. J. L.; Charlton T.; Zarbakhsh A.; Casford M. T.; Clarke S. M. Hexadecylamine Adsorption at the Iron Oxide-Oil Interface. Langmuir 2013, 29, 13735–13742. 10.1021/la4018147. 24106786 PMC3850247

[ref24] Wood M. H.; Wood T.; Welbourn R.; Poon J.; Madden D.; Clarke S. An X-ray and Neutron Reflectometry Study of Iron Corrosion in Seawater. Langmuir 2018, 34, 5990–6002. 10.1021/acs.langmuir.8b00378. 29719961

[ref25] Metelev S. V.; Pleshanov N. K.; Menelle A.; Pusenkov V. M.; Schebetov A. F.; Soroko Z. N.; Ul’yanov V. A. The study of oxidation of thin metal films by neutron reflectometry. Phys. B Condens. Matter 2001, 297, 122–125. 10.1016/S0921-4526(00)00839-5.

[ref26] Poon J.; Madden D. C.; Welbourn R. J. L.; Allen F. J.; Khan F.; Sonke H.; Clarke S. M. Corrosion inhibition of steel in seawater through surface phosphate formed from oil. Surface & Coatings Technology 2021, 410, 126970 10.1016/j.surfcoat.2021.126970.

[ref27] Feng J.; Browning J. F.; Fitzsimmons M. R.; Wang Q.; Majewski J.; Wang P.; Schefer D. W. Impact of ferromagnetism on neutron reflectometry of passivated iron. Thin Solid Films 2022, 759, 139464 10.1016/j.tsf.2022.139464.

[ref28] Ha H. M.; Fritzsche H. In-Situ Polarized Neutron Reflectometry Study of the Passive Film Growth on Fe-20Cr Alloy. J. Electrochem. Soc. 2019, 166, C3064 10.1149/2.0081911jes.

[ref29] Wood M. H.; Browning K. L.; Barker R. D.; Clarke S. M. Using Neutron Reflectometry to Discern the Structure of Fibrinogen Adsorption at the Stainless Steel/Aqueous Interface. J. Phys. Chem. B 2016, 120, 5405–5416. 10.1021/acs.jpcb.6b02341. 27244444

[ref30] Wood M. H.; Welbourn R. J. L.; Zarbakhsh A.; Gutfreund P.; Clarke S. M. Polarized Neutron Reflectometry of Nickel Corrosion Inhibitors. Langmuir 2015, 31 (25), 7062–7072. 10.1021/acs.langmuir.5b01718. 26050787

[ref31] Ha H.; Fritzsche H.; Burton G.; Ulaganathan J. Polarized Neutron Reflectometry of Metal Consumption and Passive Film Growth on Nichel Exposed to an Alkaline Deuterium Oxide (D_2_O) Solution. J. Electrochem. Soc. 2017, 164, C699 10.1149/2.0171713jes.

[ref32] Cwalina K. L.; Ha H. M.; Ott N.; Reinke P.; Birbilis N.; Scully J. R. In Operando Analysis of Pssive Film Growth on Ni-Cr and Ni-Cr-Mo Alloys in Chloride Solutions. J. Electrochem. Soc. 2019, 166, C3241–C3253. 10.1149/2.0261911jes.

[ref33] Lutton K.; Han J.; Ha H. M.; Sur D.; Romanovskaia E.; Scully J. R. Passivation of Ni-Cr and Ni-Cr-Mo Alloys in Low and High pH Sulfate Solutions. J. Electrochem. Soc. 2023, 170, 021507 10.1149/1945-7111/acb9c3.

[ref34] Welbourn R.; Truscott C.; Skoda M.; Zarbakhsh A.; Clarke S. Corrosion and inhibition of copper in hydrocarbon solution on a molecular level investigated using neutron reflectometry and XPS. Corros. Sci. 2017, 115, 68–77. 10.1016/j.corsci.2016.11.010.

[ref35] Situm A.; Bahadormanesh B.; Bannenberg L.; Ooms F.; Feltham H. A.; Popov G.; Behazin M.; Goncharova L. V.; Noël J. J. Hydrogen Absorption into Copper-Coated Titanium Measured by In Situ Neutron Reflectometry and Electrochemical Impedance Spectroscopy. J. Electrochem. Soc. 2023, 170, 041503 10.1149/1945-7111/acc763.

[ref36] Matveev V. A.; Pleshanov N. K.; Bulkin A. P.; Syromyatnikov V. G. The study of the oxidation of thin Ti films by neutron reflectometry. J. Phys. Conf. Ser. 2012, 340, 012086 10.1088/1742-6596/340/1/012086.

[ref37] Tun Z.; Noël J. J.; Shoesmith D. W. Electrochemical modifications on the surface of a Ti film. Physica B 1997, 241–243, 1107–1109. 10.1016/S0921-4526(97)00805-3.

[ref38] Tun Z.; Noël J. J.; Shoesmith D. W. Electrochemical Modification of the Passive Oxide Layer on a Ti Film Observed by In Situ Neutron Reflectometry. J. Electrochem. Soc. 1999, 146, 988–994. 10.1149/1.1391710.

[ref39] Wiesler D.; Majkrzak C. Neutron reflectometry studies of surface oxidation. Phys. B. Condens. Matter 1994, 198, 181–186. 10.1016/0921-4526(94)90156-2.

[ref40] Vezvaie M.; Noel J.; Tun Z.; Shoesmith D. Hydrogen Absorption into Titantium under Cathodic Polarization: An In-Situ Neutron Reflectometry and EIS Study. J. Electrochem. Soc. 2013, 160, C414–C422. 10.1149/2.020309jes.

[ref41] Noël J. J.; Shoesmith D. W.; Tun Z. Anodic Oxide Growth and Hydrogen Absorption on Zr in Neutron Aqueous Solution: A Comparision to Ti. J. Electrochem. Soc. 2008, 155, C444–C454. 10.1149/1.2937306.

[ref42] Tun Z.; Noël J. J.; Shoesmith D. W. Anodic oxide growth on Zr in neutral aqueous solution. Pramana J. Phys. 2008, 71, 769–776. 10.1007/s12043-008-0192-z.

[ref43] Noël J. J.; Jensen H. L.; Tun Z.; Shoesmith D. W. Electrochemical Modification of the Passive Oxide Layer on Zr-2.5Nb Observed by In Situ Neutron Reflectometry. Electrochem. Solid-State Lett. 1999, 3, 473–476. 10.1149/1.1391183.

[ref44] Hu N.; Dong X.; He X.; Borwning J. F.; Schaefer D. W. Effect of sealing on the morphology of anodized aluminum oxide. Corros. Sci. 2015, 97, 17–24. 10.1016/j.corsci.2015.03.021.

[ref45] Wang P.; Dong X.; Schaefer D. W. Structure and water-barrier properties of vanadate-based corrosion inhibitor films. Corros. Sci. 2010, 52, 943–949. 10.1016/j.corsci.2009.11.017.

[ref46] Junghans A.; Chellappa R.; Wang P.; Majewski J.; Luciano G.; Marcelli R.; Proietti E. Neutron reflectometry studies of aluminum-saline water interface under hydrostatic pressure. Corros. Sci. 2015, 90, 101–106. 10.1016/j.corsci.2014.10.001.

[ref47] Wood M. H.; Clarke S. M. Neutron Reflectometry for studying Corrosion and Corrosion inhibition. Metals 2017, 7, 304 10.3390/met7080304.

[ref48] Noel J. J. Canadian Research Combining Neutron Reflectometry and Electrochemistry. La Physique au Canada 2018, 74, 49.

[ref49] Wolff M.; Frielinghaus H.; Cárdenas M.; Gonzalez J. F.; Theis-Bröhl K.; Softwedel O.; von Klitzing R.; Pilkington G. A.; Rutland M. W.; Dahint R.; Gutfreund P. Grazing incidence neutron scattering for the study of solid-liquid interfaces, in: Encyclopedia of Solid-Liquid Interfaces; Wandelt K., Busetti G., Eds.; Elsevier: Amsterdam, Oxford, Cambridge, 2024; Vol. 1, pp 305–323.

[ref50] Payra D.; Naito M.; Fuji Y.; Yamada N. L.; Hiromoto S.; Singh A. Bioinspired adhesive polymer coatings for efficient and versatile corrosion resistance. RSC Adv. 2015, 5, 15977–15984. 10.1039/C4RA17196A.

[ref51] Watkins E. B.; Kruk I.; Majewski J.; Allred D. D. Oxide structure of air-passivated U-6Nb alloy thin films. J. Nucl. Mater. 2020, 539, 152356 10.1016/j.jnucmat.2020.152356.

[ref52] Mongstad T.; Platzer-Björkman C.; Mæhlen J. P.; Hauback B. C.; Karazhanov S. Zh.; Cousin F. Surface oxide on thin films of yttrium hydride studied by neutron reflectometry. Appl. Phys. Lett. 2012, 100, 191604 10.1063/1.4714517.

[ref53] Quadakkers W. J.; Zurek J. Oxidation in Steam and Steam/Hydrogen Environments. In Shreir’s Corrosion; Richardson J. A., Ed.; Elsevier: Amsterdam, 2010; Vol. 1, pp 407–456.

[ref54] Leygraf C.; Odnevall Wallinder I.; Tidblad J.; Graedel T. Atmospheric Corrosion, 2nd ed.; John Wiley & Sons, 2016.

[ref55] Esmaily M.; Svensson J. E.; Fajardo S.; Birbilis N.; Frankel G. S.; Virtanen S.; Arrabal R.; Thomas S.; Johansson L.-G. Fundamentals and advances in magnesium alloy corrosion. Prog. Mater. Sci. 2017, 89, 92–193. 10.1016/j.pmatsci.2017.04.011.

